# Performing masculinity, influencing health: a qualitative mixed-methods study of young Spanish men

**DOI:** 10.3402/gha.v6i0.21134

**Published:** 2013-09-16

**Authors:** Jorge Marcos Marcos, Nuria Romo Avilés, María del Río Lozano, Juan Palomares Cuadros, María del Mar García Calvente

**Affiliations:** 1 Institute for Women's and Gender Studies, University of Granada, Granada, Spain; 2 Andalusian School of Public Health, Granada, Spain; 3 Department of Didactics of M.P. and Body Expression, Faculty of Education Sciences, University of Granada, Granada, Spain

**Keywords:** gender, masculinity, men's health, young men, social theory, health inequalities, qualitative research

## Abstract

**Background:**

The literature shows how gender mandates contribute to differences in exposure and vulnerability to certain health risk factors. This paper presents the results of a study developed in the south of Spain, where research aimed at understanding men from a gender perspective is still limited.

**Objective:**

The aim of this paper is to explore the lay perceptions and meanings ascribed to the idea of masculinity, identifying ways in which gender displays are related to health.

**Design:**

The study is based on a mixed-methods data collection strategy typical of qualitative research. We performed a qualitative content analysis focused on manifest and latent content.

**Results:**

Our analysis showed that the relationship between masculinity and health was mainly defined with regard to behavioural explanations with an evident performative meaning. With regard to issues such as driving, the use of recreational drugs, aggressive behaviour, sexuality, and body image, important connections were established between manhood acts and health outcomes. Different ways of understanding and performing the male identity also emerged from the results. The findings revealed the implications of these aspects in the processes of change in the identity codes of men and women.

**Conclusions:**

The study provides insights into how the category ‘man’ is highly dependent on collective practices and performative acts. Consideration of how males perform manhood acts might be required in guidance on the development of programmes and policies aimed at addressing gender inequalities in health in a particular local context.

Nowadays, gender is considered to be largely a social construct with important direct implications for health. Thus, most social scientists agree that the definition of gender is not merely a personality trait but also a social system that restricts and influences patterned behaviour (1). This conception is widely connected with the development of explanatory models of inequalities in health research.

The study of gender inequalities received an important boost with the advent in the 1980s of the critical study of men and masculinity. This approach started as part of the feminist critique of sex role socialization theory, by not considering aspects of privilege and power within their analytical framework (2, 3). Similarly, the constructivist approaches began to establish that the constituent traits of masculinity may vary historically and culturally, which implies the discussion of multiple masculinities. This idea was primarily developed around the notion of hegemonic masculinity (4). While this has led many researchers to emphasize hegemonic masculinity as a cultural ideal, where the focus is placed on documenting the various ways of ‘being a man’, little research has been conducted on unpacking the interactional processes through which the most honoured way to be a man is locally constituted (5). That is, sight has been lost of the analysis of its consequences.

In recent decades, the understanding of gender and health issues has been widely favoured by the development of relational theory. The relational approach gives a central place to the patterned relations between men and women (and among women and men) that constitute gender as a social structure (6). Within the terms of this approach, West and Zimmerman's ‘doing gender’ perspective emphasizes the way gender is accomplished in personal interaction (7). At the same time, Butler's conceptualization of gender, from a different ontological perspective regarding the possibility of a self, has added to the discussion of ‘doing gender’ in critical ways, helping to sharpen the focus on performativity (1). Thus, taking the phenomenological perspective of ‘acts’, gender may be understood as a constructed entity that involves the repetition of acts that define shared experience as a form of ‘collective action’ (8). It is precisely this kind of gender display that has been highlighted in the literature as a characteristic element in understanding men and masculinity, describing how manhood acts as a form of subjectivity. These ‘manhood acts’ not only imply a claim to membership of the privileged gender group but also may lead to health damage (9). From a public health perspective, this damage has often been particularly associated with the behavioural aspects of ‘manhood acts’.

Scientific evidence shows that the largest gender gaps in morbidity and mortality are caused by behavioural differences between men and women (10). The literature emphasizes important differences between how men and women define and prioritize their health (11). In the case of men, one of the key factors in disease and death rates is related to their poor relationship with health care services (12, 13). Epidemiological research has consistently shown that men use health care services less than women (14) and that they are less involved in preventive and health-promoting initiatives (15). At the same time, the research has noted disparities in health literacy, in terms of how men and women access and apply the information available (16). The literature shows that many men are driven to not ask for help, as offering no sign of vulnerability is an essential trait of masculinity (6). Mental health is a significant case in point. Thus, for example, with regard to the symptoms of depression, men are often underdiagnosed and undertreated (12). This mental disorder is often associated with weakness and a loss of emotional control in men (17). At the same time, many men display their mental and emotional distress in a different way than women. This has been linked in the literature with the decline of social support networks (18), and especially with substance abuse.

In the vast majority of cultures, being considered to be ‘a man’ is a condition that must be ‘achieved’ (19). The excessive intake of recreational substances, especially alcohol, is a major cultural expression related to masculinity in the literature (20). Despite having a higher risk of falling into drug use, men of all ages perceive the risk of this activity as being significantly lower than women do (21). In parallel with drug use, masculinity has been also closely related to violent behaviour, unsafe driving, and risky sexual behaviour (22). More recently, within the studies on men's health, masculinity has been studied in relation to body image (23).

Self-confidence is strongly influenced by body image in youth populations (24). While this has been specifically studied in women, it is now also considered to be an important predictor of psychological well-being in young men (25). In Western societies, this has mainly been analysed from the perspective of the utilitarian symbolism of the muscular mesomorph. Thus, some researchers have shown a significant association between traditional masculine ideology, the pursuit of muscularity, and body image discrepancy (26). In this way, muscles become a representational sign of power and strength that, at the same time, can also lead to crash dieting, the consumption of nutritional supplements, and, in more extreme cases, the clinical symptoms of muscle dysmorphia (27).

Social expectations and stereotypes attached to gender in relation to the behaviour that is considered appropriate are factors with a great influence on the sexual behaviour of young men (28). The experience of masculinity is closely related to the exercise of sexuality (29), since heterosexuality is a key factor associated with hegemonic rules of masculinity (30). In this way, having an active sex life or ‘luck with the ladies’ may be interpreted as signs of possessing a masculine self, which has been described as being related to promiscuous behaviour and risky sexual practices (31, 32). Also, in Western societies, the vast majority of reckless drivers are young men (33). In Europe, statistics have shown that men are three times more likely to suffer an accidental death than women (34). Apart from other structural reasons, the greater tendency of men to, for example, exceed a speed limit is linked to the fact that risk is naturalized and promoted in men (35). Moreover, in all countries, men aged 15 to 45 years are both the main perpetrators and victims of homicide (36). In terms of socio-anthropological approaches, physical strength in males is at the epicentre of the association between masculinity and violence (37). The consideration of violence as a part of the global public health agenda, along with the development of ecological models of analysis, has reinforced the idea that this relationship is mainly the result of men's socialization to be dominant and the symbolic structures of the social power that perpetuate such domination (38). These last considerations gain special meaning in contexts such as the Spanish one.

After the early opening movements of the dictatorial Franco regime in the second half of the twentieth century, numerous Anglo-Saxon researchers focused on the study of society and Spanish culture. Their works constituted the basis of the study of masculinity in contemporaneous Spain (39–41). These studies, which focussed almost exclusively on rural Andalusia, described a strongly sexist masculinity where men were acknowledged as the only providers for the family unit. Men's reputations are especially described around considerations of sexuality. Thus, for example, while honour is presented as a multidimensional feature of social life, it is often associated with sexual control over women in the family, including the insistence on virginity in single people, fidelity in marriage, and the sexual abstinence of the widow. Despite the existence of these works, the study of men is still considered to be at an embryonic stage (42). This is particularly significant with regard to the study of health from a gender perspective. The vast majority of the studies that have followed this approach have focussed on comparing indicators of illness outcomes among men and women.

In Spain, the life expectancy of males is 78.5 years, 6 years shorter than that of women (43). The mortality rate from chronic diseases in people under 75 years of age is highly unfavourable to men (44) and includes more mortality from diseases that occur mainly as a result of bad health habits (such as an unbalanced diet or the use of illegal drugs, tobacco, and/or alcohol). This higher rate of mortality is also the result of external causes, such as accidents, falls, aggression, or suicides. For instance, 78.2% of deaths resulting from motor vehicle accidents occur in males, with the highest percentages being between the ages of 16 and 24 years (43). These considerations connect with the main idea of the last report by the European Commission regarding the state of men's health: there is a high level of preventable premature morbidity and mortality in men, which will be addressed only by targeted activity across their lifespans (45).

The way in which men position themselves in relation to different discourses on masculinity has important implications for the social processes of health and disease (46). In the case of young people, this theoretical basis provides a context for the idea that the higher health risks faced by men are the result of factors and behaviours that could be modified (47). Although the literature describes numerous projects and interventions designed from a gender perspective aimed at improving the health and well-being of men and women, many of these programmes have rarely shown an interest in the point of view of the men themselves (48). This emphasizes the need for an investigation of the experience of the processes of health and disease in men in different contexts. Thus, bringing out the characteristics of masculinity is to be understood as a way of contributing to the identification of local, regional, and global interactions (49). This project was designed to explore through men's narratives, from a gender perspective, the complexity of young men's worldview (their ‘lay knowledge’) and its relationship to their health; such narratives include the stories that youths express about themselves and others. The project's research focus was on the diversity of how masculinity and other practices related to health operate in the daily lives of men. In terms of men's health studies, this article takes a critical approach by carrying out an analysis of practices identified as ‘manhood acts’; that is, of what men do individually and collectively to signify their membership in the category of ‘man’ (9). This might be a useful way to reveal how men's health practices can be seen as mechanisms for constructing gender through a series of acts that are renewed, reviewed, and consolidated over time. Thus, the aim of this article is to describe the lay perceptions and meanings ascribed to the idea of masculinity, and identify ways in which young men relate gender displays to health.

## Methods

This qualitative study is based on fieldwork developed between March 2009 and April 2010 in Andalusia in southern Spain. Following intentional non-probabilistic sampling, 59 participants were recruited from youth centres and educational institutions. From the beginning, the research team tried to bring about the greatest possible control of bias when choosing sample units. This required the pursuit of multiple independent networks, unrelated to the research team. The size of the sample was strictly related to the search for different profiles. The general inclusion criteria were being a man aged 15–24 years and being born and raised in Andalusia. The sons of immigrants and young men from the Roma community were not included since the presence of specific characteristics within their cultural framework was assumed. In order to ensure the heterogeneity of the participants’ profiles, three criteria were established: age (15–17 or 18–24), origin (cities, towns, or villages), and level of education (ranging from primary education to university studies).

Nine individual interviews, eight focus group discussions, and a triangular group session were conducted. The combination of these methods enabled the socio-cultural factors and personal aspects of the object of study to be explored more deeply. The focus groups were particularly useful for identifying the lay perceptions of the expectations and signs of social desirability and behaviour that are considered appropriate. The individual interviews enabled the lay knowledge of the participants to be explored in depth. In the later part of the fieldwork, we conducted a triangular group (50). This qualitative technique, which involves three participants, was introduced because it allowed us to create a more interactive group dynamic, in which even the researcher–interviewer may have a more active role during the course of the discussion. In a triangular group, there is usually more tension between the particular positions of each participant and the common elements (or consensus view) than in an orthodox discussion group. This allowed us to confront certain emerging perspectives more exhaustively. This mixed-methods data collection strategy promoted the use of multiple information sources and different approaches to gain new insights into the object of study.

The fieldwork was developed in two consecutive and complementary stages. The first one was designed with regard to some general principles of the grounded hermeneutic approach (51). During this phase, an individual interview and a focus discussion group were carried out. With regard to the focus group, the selection of the participants was determined so that their profiles would contain certain types of heterogeneity. This criterion was established so as to contrast different positions with regard to the object of study. During this first phase, the discussion was stimulated by presenting the participants with a sequence of photographs related to the world of youth that gave them the possibility of expressing their ideas about each of them. The preliminary analysis of the data collected during the first phase of the fieldwork enabled the identification of significant topics and dominant discourses that were a basis for the elaboration of the topic guide that was developed for the second phase of the fieldwork. This guide was formulated around 10 conceptual dimensions: 1) men's typical, 2) women, 3) youth, 4) health, 5) risk, 6) violence, 7) sexuality, 8) body image, 9) feelings, and 10) homosexuality. During the second phase of the fieldwork, eight semi-structured interviews, seven focus discussion groups, and a triangular group session were carried out. In this phase, the discussion groups were formed so that they would involve homogeneous profiles. The completion of the data collection process was determined according to the principle of saturation (52). All the interviews were recorded and transcribed literally by a member of the research team. The transcriptions were completed using the interviewer's field notes.

We carried out a qualitative content analysis. Because of the aims and theoretical perspective of the study, the research team decided from the beginning that the analysis process would extract not only the manifest content but also the underlying meanings (53). Thus, after a general reading of the transcriptions, the text about the men's behaviour as being gendered was extracted and brought together into one text, which constituted the unit of analysis. Later, the text was divided into units of meaning that were condensed. At the same time, the condensed meaning units were abstracted and labelled with a code. The dimensions and sub-dimensions proposed for the analysis were applied by different members of the research team so that there was agreement upon their definition and how they would be applied, thus lending reliability to the analysis process. The conceptual classification of codes and categories was constructed by using both a deductive research approach and an inductive procedure. Thus, the process of analysis began with a predetermined list of topics to be explored and a category system that had previously been defined after the first phase of the fieldwork. In the same way, any concepts, categories, or sub-categories that emerged from the examination of the data during the analysis process were integrated into the analysis.

The revision of concepts and categories and the encoding process were carried out by the main researcher in collaboration with a specialist in qualitative methodology external to the research. This procedure was not only part of the triangulation process but also a way of adding higher confidence to the analysis. The texts were sorted into seven conceptual areas, which constituted the basis of the manifest content: 1) femininity versus masculinity, 2) health and well-being, 3) sexuality, 4) body image, 5) substance abuse, 6) driving, and 7) violence. During the last phase of the analysis process, these elements were integrated into two themes: 1) representing power: body image and violence; and 2) typically masculine: driving, drugs, and sex.

To facilitate the dual process of interpretation (manifest and latent content), the analysis was developed with the support of the QSR NVivo 8 program and took a hermeneutic approach involving the following steps: 1) a general reading of the texts to obtain a sense of the whole; 2) the review of any topics and examples arising, with an analysis of shared and unshared content and their significance in light of each profile; 3) the identification of interrelated topics; and 4) an analysis of the ways in which gender identity was related to life experiences that influence health (54). In this sense, the research team focused on manhood acts – the ways to signify masculine selves. Thus, we interpreted the data by looking for what participants said about how men learned to perform “manhood acts,” how and why these acts can vary, and how they can reproduce gender inequalities.

The research protocol that forms the basis of this article was approved by an internal commission established by the European Public Health Master ‘Europubhealth’. According to the current Spanish law (Organic Law 15/1999 of 13 December), the personal data provided by the participants were stored and kept in a safe file, and real names were replaced by fictitious ones. Participation was voluntary, and the participants received information about the objectives of the study as well as about the institutions involved. All participants signed an informed consent form. Parents or guardians’ consent was required for underage participants.

## Results

### Representing power: body image and violence

Body image and the aggressive use of force were aspects that were mainly linked to the social representation of power in men's actions. These were related to the perception of the body as a source for signifying manhood, emphasizing it as a tool for control, dominance, and activity. Men are socialized to use the body to symbolize manhood. In this process, according to the participants, the practice of sport was considered central. From childhood, men are more exposed to playing competitive games based on physical performance, which encourages them to display fortitude, control the experience of physical pain, and endure intense effort. For instance, in the specific case of football, ‘toughness’, ‘aggressiveness’, and ‘courage’ were words used to justify why a sport was seen as being more suitable for men.

CARLOS: If you are playing football and a player kicks you and you complain, somebody may tell you that this is a man's game. But the truth is that when you are participating in the competition you have to fight aggressively for the ball, because that can influence the final result. When somebody kicks you hard, rivalry arises. (Individual interview, town, 22 years old, enrolled at university)

These traits were considered to be factors with important health implications, especially in terms of risk-taking behaviour and the gender differences involved in coping with illness. With regard to the latter, reluctant attitudes towards the use of medicines and medical consultation were recognized by participants as behaviour traditionally embedded in ideologies of masculinity, which are related to physical and emotional toughness. Although some participants admitted to going to the doctor every time they perceived any anomaly in their health status, a large majority said that they only went ‘when it is really necessary’. This was also an attitude that the participants associated with the idea of being a responsible user of health care services. The following excerpt not only is a good example of this view but also suggests how men's underutilisation of medical services may be considered a ‘manhood act’.

PEDRO: All the girls have a little bag full [of] medicines at home. And as soon as they have the slightest headache, they take a tablet; a stomach ache, one pill; another sort of pain, they go to the doctor. And I have to be really sick to go to the doctor.… it's also true that if a friend [boy] goes to the doctor every day, we say: ‘What's wrong with this guy, he's always at the doctor! He gets sick so easily!’ (Group interview, city, 20–23 years old, enrolled at university)

Disruptive behaviour and episodes of vandalism or physical aggression were especially considered to be recurrent actions by men. This was a generalized perception of the participants in the study, including those who, like Ricardo, emphasized the need for speaking about plural masculinities.

RICARDO: You cannot really lump all men together. There are thousands of different types of men and each of them is different from the other. The only thing that differentiates us from women is physical appearance, but regarding attitudes, that depends on education. A man can be as crass as a woman. But it is true that I have seen boys jumping on cars or burning containers and I still haven't seen a woman doing that. I can't find an explanation as to why men have to fight and women don't. It's an attempt to show that I'm more than you are. For men, he who is the stronger is more so than the other. (Individual interview, town, 19 years old, enrolled at university)

During this study, references to violence against women were minimal. When they were made, the participants took positions of rejecting this type of violence. The more widely reported forms of violence were those that were established among men. Although most of the participants argued against the use of violence, they all declared that at some point they had witnessed a fight between men or had been directly involved in it. Violence was mainly described as a formula for proving superiority and gaining respect. Thus, the aggressive use of force reinforced the notion that to be a man is to be dominant. In this sense, violent behaviour was also connected with the idea of protection, widely considered to be a part of the masculine essence by the participants.

ÓSCAR: Men fight more often. I might have seen a few girls pulling each other's hair, but that's it. But men fighting …. As for me, when I get into fights I don't stop until I see the other guy bleeding. Why? Because my balls are bigger than his. (Group interview, village, 15–17 years old, vocational training)

RAÚL: I think we have the obligation to provide women with one thing: security. For instance, if I had a daughter with a boyfriend, and for any reason they came back home saying that they were mugged and I found out that he didn't do anything, he'll be in big trouble. He's the one who has to come back with a broken jaw. And the other way round too. If I found out that something happened to her and that my son did nothing! (Individual interview, village, 23 years old, musician)

Data suggest that concern about body image is a growing phenomenon among men within the context of the study. Whether for reasons of hygiene or aesthetics, references to practices such as depilation were common. Although these are still considered minority practices, the participants expressed the view that these customs are part of the social embodiment of contemporaneous masculinity. Less standardized practices were also identified, such as the use of make-up as a way to increase the possibilities of seduction. Thus, ‘feminine’ behaviour, such as cosmetic use, is acceptable by men if it has a performative function related to heterosexual conquest.

GUSTAVO: Some of my friends get waxed and use make-up. I see it as faggoty, though I'm talking about guys who like girls! They feel more handsome and that it attracts girls. (Individual interview, town, 21 years old, primary studies, currently unemployed)

However, our findings indicate that when men are interested in improving their body image, they focus mainly on muscle development. Muscles were considered a key element within current masculine subjectivity, especially related by participants as an element of the representation of power and strength and characterized as a symbol of seduction. This was linked by participants to the perception of an increasingly widespread use of nutritional ergogenic aids among young men. The use of protein supplements was particularly reported. Although some participants expressed opposition to the use of this type of dietary supplement, the vast majority of participants described these products as harmless, and they considered professional consultation and/or prescription to be unnecessary. Participants also reported cases in which, as a way of achieving the desired muscle growth, some men had turned to the use of anabolic steroids. Although the participants considered the number of young men who accessed these products to be low, the lay perceptions that emerged from the analysis are of great interest. In spite of being products that can only be purchased legally under medical prescription, the perception of ease of access to them in gyms or shops that specialize in sports nutrition was general across our informants.

JAVIER: Gyms supply proteins. There are many shops that sell them too. Proteins are legal, not anabolic steroids or any other chemical substances, but if you wanted them, you could get them easily. You can get them from your monitor in the gym. I'm sure he is a wrestler and he deals with this stuff. It is the same as if you are a police officer who knows where to go to get cocaine; isn't it? (Group interview, city, 20–23 years old, enrolled at university)

### Typically masculine: driving, drugs, and sex

During this research, the risks associated with driving were those most directly related to health. ‘Caution’ was a term that was widely used to define women's attitudes toward driving motor vehicles. Exceeding speed limits, driving under the influence of alcohol or (in the case of motorcycles) without a helmet, and showing skills like driving on a single wheel were described as typically male forms of behaviour; that is, forms of social representation of manhood through which men seek attention and recognition from their peer group.

CARLOS: We do wheelies because someone always starts – first to show off in front of us and then, if there are any girls around, to show off in front of them. It is a way of proving that you are the fittest or the cockiest; the most daring, because you don't ride like the rest. If everybody did it, it wouldn't be cool. It's like you have to try and do something different. (Individual interview, town, 22 years old, enrolled at university)

When participants referred to the consumption of recreational substances, their statements were dominated by references to alcohol, and more specifically to *botellón*.^1^ All the participants in the study, including minors, referred to *botellón* as being a common practice in their leisure time. Some young men showed resistance to traditional gender traits, positioning themselves as being in favour of responsible use and/or expressing awareness of the health risks of alcohol abuse; for others, those risks were not so obvious. Thus, heavy drinking per se was not considered risky. It was only seen as such when combined with other risky forms of behaviour.

FERNADO: I see that all of us got drunk but that's not risky, is it? Well, it is risky if you are drunk and you are riding a motorbike, which is quite dangerous. (Group interview, town, 15–17 years old, enrolled at high school)

Most of the participants acknowledged that alcohol abuse is still judged by society differently for men and women. Despite this double standard for alcohol use, the data indicated the existence of an established perception among participants: girls have become involved in a ‘masculinization’ process regarding alcohol abuse at weekends. On the other hand, binge drinking is a practice rooted in the process of construction of the meaning of ‘being a man’. It is seen as a practice symbolizing manhood; a performance of *until the body gives up* closely related to the process of achieving a feeling of belonging to a peer group. In some cases, as described by Raúl, the heavy consumption of alcohol as a ‘manhood act’ becomes a ‘revised’ gendered act over time.

RAÚL: When you were younger and went to a ‘botellón’ on an empty stomach you'd have seven or eight drinks. That's definitely no good for your health and you know it, but whether you want to or not, you do it because everybody does. I never drank as much as the others did because I didn't like it, but I drank because everybody else did. Now, when we go to play football, everybody drinks a pint of beer. I don't. I drink a Coca-Cola. Still today everyone goes: ‘Come on. You're a guy!’ Now I don't care, but when I was 16 …. (Individual interviews, village, 23 years old, musician)

With regard to expressions like *we men are more vicious*, the participants revealed their belief that the use of other recreational drugs is also a habit that is more prevalent among men. Of special significance were the references made to marijuana and hashish consumption. These recreational substances were considered to be in widespread use among young Andalusian men. Participants also expressed the opinion that the harmful effects of these substances were not significant. The idea of risk with regard to the use of psychoactive substances was particularly associated with ‘synthetic drugs’. Overall, the perception of participants was that the use of these types of substances was increasing. These drugs were connected to having a lack of inhibition and were described as being facilitators of casual relationships. The only informant who openly expressed having used the drug ecstasy throughout this research declared that he had done it as a way of experiencing new feelings during sex.

Sexuality was considered by the participants as being of vital importance to the construction of male subjectivity. Participants focused their attention on the tensions between the cultural and biological explanations of men's behaviour. The core element of the discussion revolved around the sexual appetite. Those who believed that sexual desire is greater in men resorted to arguments with a biological background by using concepts such as *instinct*, *essence*, or *nature*. However, other participants considered that if such beliefs are socially established, it is because men tend to be expected to be more open about their sexual desires. While for a man such openness is considered to be a sign of manhood, women tend to care more about such conduct since it may lead them to be judged more negatively by society in terms of their morality. Although our findings show that many girls no longer play the passive role that traditionally characterized them when establishing a relationship, the participants expressed the view that they are generally the ones who take the initiative, or they are aware that girls pretend not to do so in order that they are not labelled as ‘easy’.

LUIS: I think we have a sexual drive, I don't know if it's because of our age or because we are men, it's just not normal!

JUAN: But don't get me wrong; it's both men and women, isn't it? What happens is that women are more reserved than we are. We show it more openly. We're cheekier and chicks know it. (Focus group, 19–21 years old, enrolled at university)

The results point to a widespread lay perception that men live their sexuality in a less emotional and more genital-based way. This was frequently related to other perceptions: 1) the importance of satisfying the sexual expectations of a partner (either stable or occasional), and 2) the greater promiscuity of men. With regard to this, different ways of living one's sexuality and characterizing masculinity were expressed. The results highlight the need to consider multiple forms of masculinity in relation to sexual life. In fact, some participants distanced themselves from ‘masculine’ predatory heterosexuality, while others contributed to reinforcing it. These two opposing positions can be deduced from the words of Marcos, Víctor, and Pablo:

MARCOS: I think sex is the same as eating, it's a basic need. Why not do it if the occasion arises one night with a girl that you know wants what you want?

VÍCTOR: I don't like to sleep with a different girl every night. It's as if it were a jacket instead of a chick. It might sound a bit backwards, but I have my morals. When I was 16 I, like my friends, used to go for the first one that came by. Now I look for other things in a woman, not just sex. (Triangular group, 19–22, different educational background)

PABLO: I had a girlfriend until about three weeks ago, and I wasn't going around saying: ‘I screw her this way and that!’ But when I go out and get laid, the first thing I think of is to call a buddy to tell him about it. It's like if you don't tell anyone, it's as if it didn't happen. (Individual interview, 20 years old, electrician)

From a sexual point of view, all these considerations around men's identity were closely connected to issues related to risky sexual behaviour and the possible power relations between males and females. Although some participants mentioned the prevention of sexually transmitted diseases, the idea of risk in this field was mainly associated with potential pregnancy. Likewise, the debate about whether or not to use a condom was usually linked to the distinction between being a stable partner or an occasional partner. Not only were condoms associated with a loss of sensitivity, but also not using them was associated with a leap of confidence in an intimate relationship. Having unprotected sex was described as a sign of manhood among the peer group, and this was sometimes seen as an added motivation for not using a condom:

GUILLERMO: Men also use this behaviour as a way of asserting masculine superiority. If I tell some friends that I have never done it [without a condom,] they feel ‘superior’, and they boast that they do it without a condom. That's the reason for them to take the initiative to start doing it without a condom. (Individual interview, 24 years old, enrolled at university)

Moreover, during this work, most participants showed that with an occasional partner they tend to use a condom, but many of them (including some minors) admitted to having had unprotected sex at some point.

DAVID: With my regular partner, I have done it without a condom. Well, I've also done it with a girl I met on a trip. We did it without a condom because she was taking those pills every day. And I believed that. It seemed like the girl came from a good family. (Focus group, 18–22 years old, vocational training)

## Discussion

This article, which is based on a qualitative study of young Andalusian men, has explored the lay perceptions and meanings ascribed to the idea of masculinity, identifying ways in which gender practices are related to health. The gender differences in health outcomes between men and women are mainly linked to men's display patterns. Thus, a lower level of use of both health services and pharmaceutical drugs, as well as a greater tendency to practise physical activities and sports, were some of the core aspects of the behaviour of the participating men. The relationship between masculinity and health is mainly defined according to behavioural explanations with a clear performative character. Ideas about reckless driving and violent behaviour established important connections between the perceptions of health and ‘manhood acts’. In addition, different ways of understanding and performing men's identity were revealed through the data gathered on the attitudes to practices regarding male body image, the exercise of sexuality, and the use of recreational substances. There was also a clear relationship to the processes of change in the identity codes of men and women. Such changes appeared to have health implications and some significance in terms of the development of gender inequalities.

Our findings are consistent with those studies that identify muscle development as the main source of concern about body image among men (56). The literature shows that through muscle development, the male body image becomes a symbolic asset in the social representation of the hegemonic traits of masculinity (57). With regard to our findings, this is of special significance when aggressive behaviour is associated with a perception that supremacy is related to the tendency to demonstrate personal strength and protective ability. Our results also emphasize the importance of peer groups, and above all women, in this social representation process. For those who are labelled as ‘winners’, especially in practices of seduction and conquest, the mesomorphic body type is a source of respect and prestige, which has health implications for men. In this case, the findings have called attention to the increased consumption of nutritional ergogenic aids, particularly protein supplements and what have in recent years commonly come to be called *fat burners*. The use of anabolic steroids was also identified. This synthetic substance, designed to imitate the effects of testosterone, is associated with considerable risks for the reproductive and cardiovascular systems (58). The literature also describes how the difficulty of legal access to these substances often leads to the use of the black market, where products are purchased without any health and safety assessment (59). This is an issue of great interest in the public health field that has been barely studied. This is especially true if, as our findings indicate, easy access to this type of substance is widely assumed.

If gender is a historically and culturally constructed social category, and risk taking is understood as an element in this process, the use of substances should be a core aspect of this. This aspect has traditionally been emphasized as a trait that is close to the essence of what is considered masculine, especially in relation to alcohol consumption (60). Our findings show that the health risks arising from alcohol use are related not only to excesses but also to the sexual abuses and violent forms of behaviour that can ensue. Likewise, the results reveal the inverse functionality of alcohol to the identities of men and women. In Spain, some of the recent literature has shown how men interpret the assumption by women of lifestyles that have been considered traditionally more appropriate for men, such as ways of achieving a higher level of social equality and personal freedom (61). Recent studies have demonstrated that the traditional links between gender and alcohol consumption may be under revision (60, 62). In this way, while this process of the ‘behavioural migration’ of women is especially noticeable when it comes to alcohol use (63), the increase in the use of other psychoactive substances is another issue of interest in our context (64). Thus, for women, the development of consumption patterns similar to those of men could be considered to be a way of undoing gender, or of changing social interactions (65) by creating new forms of femininity that involve complicity with ‘manhood acts’. Using this approach, the dichotomy between doing and undoing gender could involve carrying out practices that for some people indicate perpetuating values and dominant beliefs, while others take them as a basis for contributing to their transgressions. According to our findings, the functioning of this gender differential is present not only in relation to the use of recreational substances but also in a proactive role in sex.

The study has identified the common lay perceptions of a type of sexuality in which men have less need for emotional intimacy. It has also indicated that sending signs of willingness to have sex is a ‘manhood act’ rooted in what is believed to be expected from a man. While the participants in the study have described a new form of femininity characterized by the expression of a more explicit and active form of sexual identity, the literature shows that when men want to regain the initiative and/or preserve their dominant position they often try to limit the sexual advances of women (66). This is another relevant issue with health implications for future research in this area, including research into the perspectives of the subjects involved – women and men alike. The literature highlights the influence of this on the social imagination, particularly with regard to the fact that men have generally been represented as being more proactive, that behaviour such as infidelity has been considered part of their biological essence, and that having unprotected sex is categorized as a ‘manhood act’ (9). These are practices that could be playing an important role as performative acts in the identity reaffirmation of heterosexual men, and in the development of power relations between males and females.

## Conclusion

This qualitative exploratory study provides information on the knowledge, attitudes, and practices that characterize young men's identities in the current Spanish context. The findings show that the category ‘man’ as a social construct is highly dependent on collective practice. Thus, focusing on ‘manhood acts’ from the point of view of their performative character provides the key to understanding men's health in a more holistic way, and it also helps to explain the inequalities in health between women and men. Similarly, information about forms of behaviour and ways of signifying masculinity that challenge the traditional codes has emerged. More research is needed, however, into how these non-hegemonic modes develop and operate in society, particularly with regard to how some young men establish a masculine identity while still rejecting ‘typically male’ stoic attitudes, dominant practices, and risky behaviour. In other words, their thoughts and practices do not act as mechanisms that signify and reproduce traditional manhood. This clearly also has implications for health outcomes in men and women.

The ‘manhood act’ perspective might provide a useful framework for the analysis of gender inequalities in health, using a life course approach. Our article highlights the importance of social representation in the public sphere in shaping masculinity. The way in which power relations are shaped and are at the root of gender inequalities has been described extensively in the literature; this reinforces the relevance of analysing not only the acts of the ‘builders of masculinity’ in a given context, but also how the notions and behaviours that constitute the meanings of masculinity are subject to change over time. From the perspective of a critical explanatory framework of men's health that is focused on ‘manhood acts’, the joint consideration of ‘practice’ and ‘performativity’ – in the terms described by Pierre Bourdieu and Judith Butler – may reinforce the relevance of studying how daily acts are gendered and socially located, and this could provide new insights in future research into how inequalities in health are embodied by men and women within a society. Some recent literature has emphasized that a key factor in advancing the understanding of men's health is located in the development of gendered epidemiology, so that through this we can begin to unpack how men's health practices can be mechanisms for ‘doing gender’ (67). With regard to this, our findings might improve the interpretation of epidemiological data in our particular context. In addition, the approach taken in this article, by directing attention to how men perform ‘manhood acts’, may facilitate awareness both of the complexity of the links between men, masculinity, and health, and of the norms, power dynamics, and practices that perpetuate health inequalities. We consider that the health sector can play a key role in the processes of social engineering to address these disparities. In this sense, it is important that health professionals are as responsive to the singular needs of men (and women) as they are to the gender-based barriers faced by them with regard to their health. In the same way, our findings suggest that although the practitioners have an important role in promoting male access and use of health care services, this engagement should go beyond simply giving attention to preventive physical health and lifestyle advice. It should address wider issues related to gender norms and social practices that perpetuate inequalities; that is, promoting responsible fathering and parenting, engaging men as caregivers, addressing gender-based violence, and so on. Thus, in order to increase the effectiveness of programmes and interventions that promote the questioning of attitudes and behaviours related to ‘unhealthy masculinity’, policies must facilitate the integrated development of gender that is mainstreamed into different social settings, without forgetting the health care system.

## Footnotes

1 *Botellón* refers to a social phenomenon that has become popular in public spaces in Spain since the end of the twentieth century. This consists of mass meetings of young people, mainly to chat while consuming some type of drink (55).
